# Enhanced stem cell image segmentation by leveraging visual processing mechanisms

**DOI:** 10.3389/fbioe.2025.1699691

**Published:** 2025-12-03

**Authors:** Zheng-mian Zhang, Hai-jun Wang, Xiao Liang, Zhi-yu Liu, Jun-yuan Hu, Gang An, Mu-yun Liu

**Affiliations:** 1 Fujian Maternity and Child Health Hospital College of Clinical Medicine for Obstetrics & Gynecology and Pediatrics, Fujian Medical University, Fuzhou, Fujian, China; 2 Shenzhen Cellauto Automation Co., Ltd., Shenzhen, Guangdong, China; 3 National Engineering Research Center of Foundational Technologies for CGT Industry, Shenzhen, Guangdong, China; 4 Harbin Beike Health Technology Co., Ltd., Harbin, Heilongjiang, China

**Keywords:** image segmentation, phase contrast microscope, stem cell image processing, visual information cognitive mechanism, confluency

## Abstract

**Background:**

This study aims to investigate the application of visual information processing mechanisms in the segmentation of stem cell (SC) images. The cognitive principles underlying visual information processing were analyzed, and the limitations of conventional segmentation methods were evaluated using phase-contrast microscopy images of stem cells.

**Methods:**

An optimized segmentation method incorporating halo correction was developed to address the limitations of traditional approaches. The performance of the proposed method was experimentally validated and compared with existing techniques.

**Results:**

The proposed method achieved segmentation accuracy, recall, precision, and F1-score values of 96.5%, 94.9%, 91.4%, and 93.9%, respectively, outperforming existing approaches. Additionally, the confluency error on the Human Mesenchymal Stem Cells dataset and the C2C12 dataset was 0.07 and 0.05, respectively, indicating superior performance compared to equivalent methods.

**Conclusion:**

The findings demonstrate that the proposed method offers enhanced efficacy for stem cell image segmentation tasks.

## Highlights

- This study explores visual info processing mechanisms in stem cell image segmentation, addressing flaws of conventional methods via phase - contrast microscopy images of stem cells.
- An optimized segmentation method with halo correction is proposed, achieving high accuracy, recall, precision, and F1 - score in stem cell images, surpassing existing approaches.
- Experiments show the method has lower confluency error on hMSCs and C2C12 datasets than equivalent ones, indicating its better performance in stem cell image segmentation tasks.

## 1 Background

Single-cell analysis based on microscopic imaging represents a critical and rapidly advancing area within the life sciences, particularly in applications such as cell clustering, tumor microenvironment quantification, and disease diagnosis (Waisman et al., 2021; Zhu et al., 2021; Moen et al., 2019). Stem cell (SC) image analysis using computer vision techniques is typically formulated as an instance segmentation task. However, the acquisition of large volumes of annotated data remains a significant challenge, as manual labeling of individual cells in microscopic images is both time-consuming and labor-intensive, often requiring the involvement of domain experts.

Accurate monitoring of the SC differentiation process is essential for the advancement of regenerative medicine strategies (Lien et al., 2023). Traditional observational approaches generally involve manual microscopy, which imposes substantial procedural complexity and workload (Orita et al., 2019). Furthermore, to enhance visualization of SC division and proliferation, staining and fluorescent labeling are commonly used—procedures that may adversely affect cell viability (Gomariz et al., 2020; Lee et al., 2021). Thus, developing methodologies that allow for effective analysis of SC behavior while preserving cellular activity remains a key objective.

The emergence of computer vision technologies has facilitated more quantitative and automated analyses of SC images (Patel et al., 2020). However, images acquired through microscopy, particularly those obtained via phase-contrast techniques, differ significantly from standard digital photographs. These images often contain substantial noise, artifacts, shadowing, and other distortions resulting from the limitations of imaging equipment, which complicate the extraction of cellular boundaries and edge features (Ma et al., 2019; Chumakova et al., 2019). Automated segmentation of cells in fluorescent microscopy images also presents difficulties due to issues such as autofluorescence, which produces irregular background intensity and hinders the separation of foreground and background regions (Watanabe et al., 2020). Furthermore, during SC growth and proliferation, overlapping and adhesion among cells introduces additional segmentation challenges (Wu et al., 2021; Edlund et al., 2021). Consequently, conventional digital image processing techniques are often insufficient for such tasks, necessitating the development of specialized methods (Schwendy et al., 2020).

In light of advancements in the understanding of visual information processing, particularly hierarchical cognitive mechanisms, new approaches to SC imaging have emerged. This study examined visual information processing frameworks and examined the limitations of existing segmentation techniques applied to SC images captured using phase-contrast microscopy. An optimized image segmentation method incorporating halo correction was proposed and evaluated. Validation experiments demonstrated that the proposed method outperformed comparable approaches in segmentation accuracy, indicating its potential use in SC image processing.

## 2 Research methodology

### 2.1 Visual information cognitive mechanism

Scientists have established that more than half of the human cortex is involved in visual processing. Within the visual neural regions, information related to direction, movement, and brightness is extracted and subsequently transmitted to higher-order receptive fields. These higher regions are characterized by increasingly larger receptive fields. Visual information is processed in a hierarchical and layered manner, where each layer builds upon the previous without interference, allowing for efficient transmission.

Approximately 80%–90% of external information is conveyed to the brain via the visual system, making it the most significant sensory modality. This information includes brightness, edges, shapes, motion, color, and depth. However, only a small fraction of the visual input reaches the visual cortex, as most is filtered out—a phenomenon referred to as “degradation” during visual transmission.

Despite ongoing challenges in understanding the hierarchical visual processing of the brain, numerous models inspired by this mechanism have been developed in the field of computer vision. In the context of this study, “this mechanism” specifically refers to the human visual system’s inherent tendency to prioritize edge and boundary feature perception, as well as its hierarchical information integration logic. Drawing on this core characteristic, we optimized the two key modules of our proposed image processing method: for the filtering module, we enhanced the extraction of cell boundary features (consistent with the human visual system’s focus on edges); for the halo correction module, we preserved detailed structural information while eliminating artifacts (aligning with the brain’s hierarchical processing principle of “retaining effective information and filtering redundancy”). These models have notably advanced applications such as cellular image analysis.

### 2.2 Adaptive threshold SC image segmentation based on visual information

The phase contrast microscope is capable of converting subtle phase shifts in light—caused by the specimen—into amplitude variations that are detectable by the human eye or a camera. However, due to its imaging principles, a halo artifact commonly forms around the specimen, which adversely affects the precision of SC image segmentation. This type of microscope is specifically designed for phase contrast imaging, also referred to as Zernike phase contrast, a technique that enhances the contrast of transparent specimens by exploiting the physical principle of phase differences.

This approach is primarily used for observing unstained, live cells and other transparent samples, which appear nearly invisible under bright-field microscopy. The use of dyes in such cases is generally avoided, as it can compromise cell viability. Through phase contrast imaging, variations in material composition and thickness are translated into contrast differences, thereby making previously indistinct structural details visible.

To address the challenges associated with halo artifacts in SC image segmentation, a method incorporating halo correction has demonstrated improved performance in phase contrast microscopy. However, the overall segmentation accuracy remains dependent on the effectiveness of the initial threshold-based coarse segmentation, underscoring the importance of selecting an appropriate threshold during the preprocessing phase.

**FIGURE 1:**
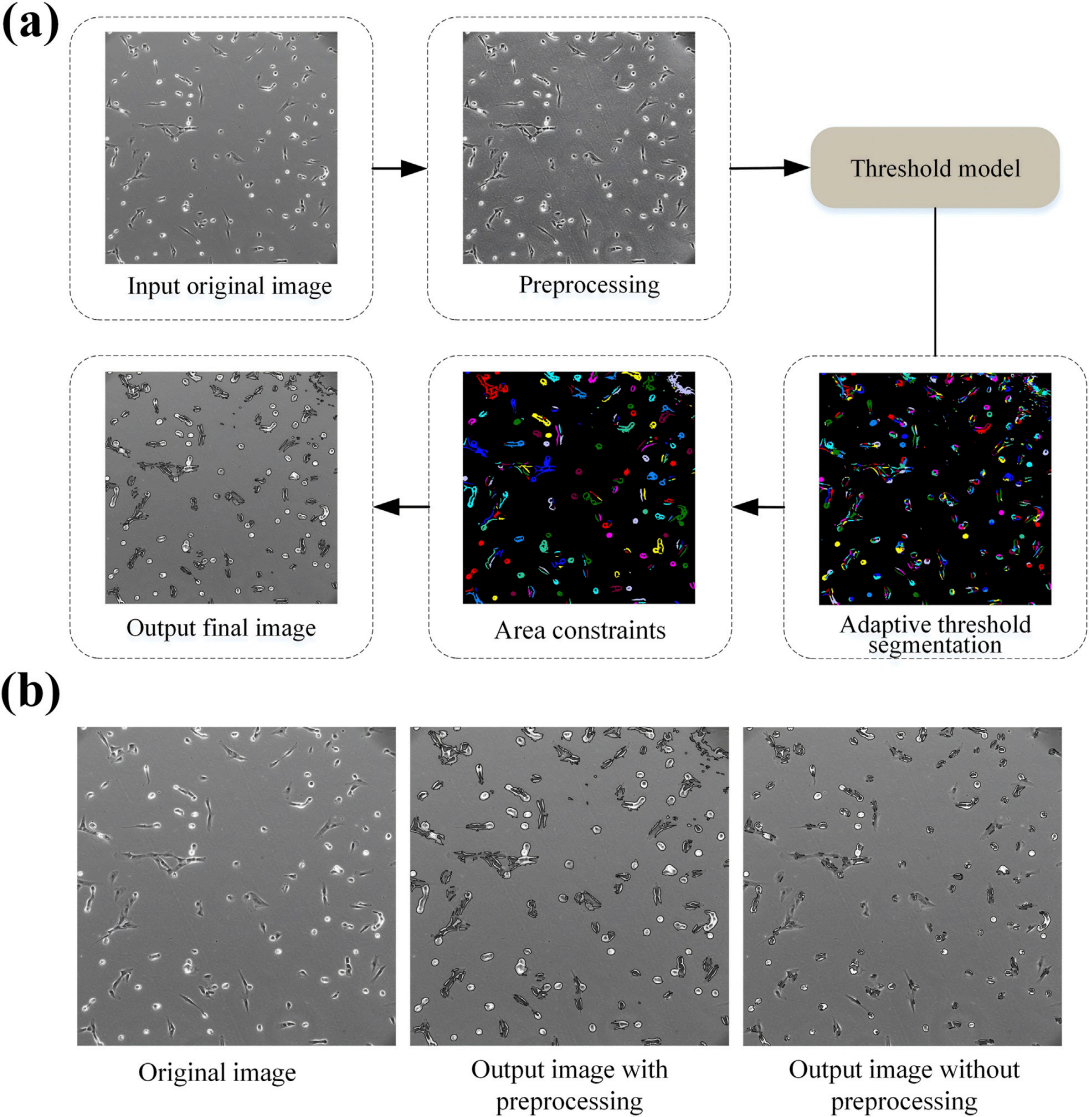
**(a)** Flow chart illustrating a threshold model process: input original image undergoes preprocessing, followed by adaptive threshold segmentation with area constraints, resulting in the output final image. **(b)** Three images show the original image, output image with preprocessing, and output image without preprocessing, respectively.

In this study, a segmentation approach combining adaptive thresholding with halo correction is proposed. This method consists of three primary stages: preprocessing, adaptive threshold segmentation, and area-based constraint application. The procedural flow is depicted in Figure 1a.

When SCs are cultured as a monolayer *in vitro*, the contrast between the cell regions and the background tends to be low, and pixel-level overlaps are frequently encountered. Therefore, enhancing image contrast and detecting variations in high-intensity pixel regions are essential steps. Initially, a gray-scale transformation is applied to the input image, denoted as $f\left(x,y\right)$ , and the gray-scale image $I\left(x,y\right)$ is obtained. Then, the local image is defined as C (x, y) and it is obtained by convolution of the gray image $I\left(x,y\right)$ using a filtering window *w*, as expressed by the following Equation 1:

$$C=\sqrt{w^{*}I^{2}‐{\left(w^{*}I\right)}^{2}}/\left(w^{*}I\right)$$ (1)

Filtering window *w* is a two-dimensional convolution kernel for image preprocessing. Specifically, *w* is a 3 × 3 square Gaussian convolution kernel that slides over the grayscale image and performs convolution operations to extract local features, thereby suppressing noise while preserving cell boundary information.* represents the convolution operator. The aforementioned preprocessing effectively addresses issues related to uneven grayscale distribution and low contrast between cells and the background in SC images. The processing time is very short, approximately 1–3 s per image, depending on the number of cells in the image. All speed tests were conducted on a standard desktop computer with an Intel Core i7-10700K CPU and 32 GB RAM (without GPU acceleration), which reflects the lightweight advantage of the proposed traditional image processing method. This enhancement serves as a critical preparatory step for subsequent image segmentation. Following preprocessing, a coarse threshold segmentation is performed.

Traditional thresholding methods are often inadequate in capturing the comprehensive statistical characteristics of image intensity distributions. To overcome this limitation, an adaptive thresholding model is introduced. The implementation of this model involves two main stages: feature extraction and threshold model construction.

In the feature extraction phase, three key features are identified from the dataset: the rate of rise (β), peak strength $M_{p}$ , and the maximum rate of rise $\beta_{\max}$ . The rate of rise reflects the slope leading to the peak in the grayscale distribution curve. Since pixel intensity values for cellular regions and background areas can overlap—particularly in cases where background regions are darker—these features are essential in distinguishing target regions from the background. Specifically, the rate of rise is instrumental in enhancing discrimination accuracy between cell structures and background noise.

Then, the model estimation is conducted according to the extracted features. Equation 2 presents the threshold estimation process.

$$T=M_{p}+\frac{kM_{p}^{2}}{255}\left(\frac{\beta }{l\beta_{u}}‐1\right)$$ (2)

$M_{p}$ serves as a reference position in the adaptive thresholding model. The variable k represents the parameters of the adjustment model in different datasets, and l = 2 is defined as a constant. The parameter $\beta_{u}$ represents the maximum rate of rise within the estimated dataset.

**FIGURE 2:**
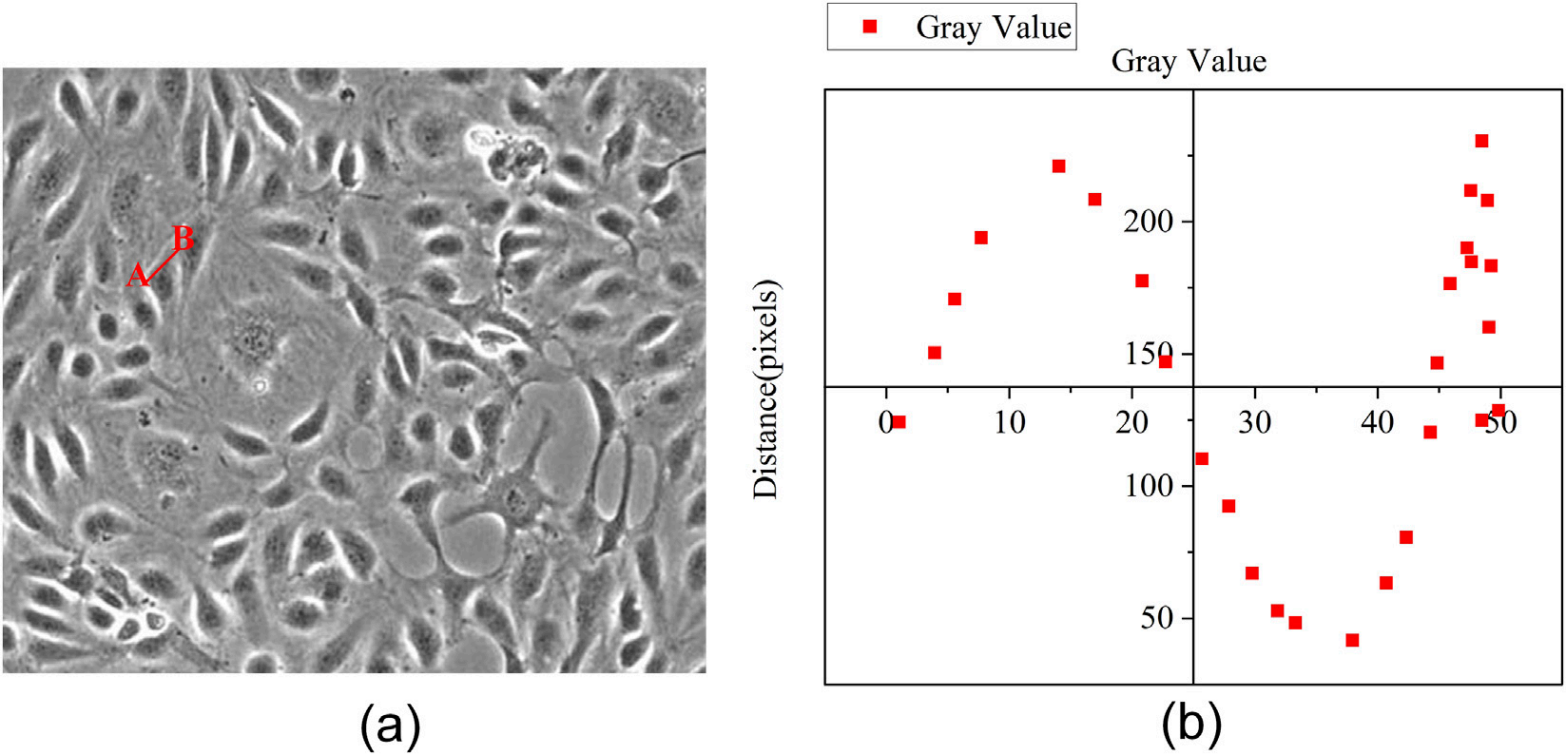
**(a)** Two red dots, A and B, were selected in the microscopic image of cells. **(b)** A pixel intensity distribution curve is generated by traversing from point A to point B.

In phase-contrast microscopy images of SCs, the presence of halos around cellular structures often introduces significant interference, compromising the accuracy of image analysis. Therefore, halo correction is necessary. The process is depicted in Figure 2.

A pixel intensity distribution curve is generated by traversing from point A to point B according to this figure. During this traversal—from the initial estimated contour to the actual cell boundary—the pixel intensity within the halo region gradually increases. As the contour approaches the true cell boundary, a decline in pixel intensity is observed in the direction of the stem cell. This pattern forms the basis for the halo correction algorithm, which begins with the edge contour derived from adaptive threshold segmentation as the initial reference set. Gradient values are then computed in eight directions: top, bottom, left, right, top-left, bottom-left, top-right, and bottom-right. The algorithm subsequently assesses if the gradient is negative. If a negative gradient is detected, the iteration is terminated; otherwise, the contour set is updated until an accurate cell boundary is obtained.

To evaluate the performance of the proposed segmentation approach, two datasets were used: the C2C12 dataset and the Human Mesenchymal Stem Cells (hMSCs) dataset. Each dataset contains 50 images, which can be publicly accessed on the website ( https://figshare.com/articles/dataset/Enhanced_Stem_Cell_Image_Segmentation_by_Leveraging_Visual_Processing_Mechanisms/30486818 ). The C2C12 dataset contains images of the C2C12 cell line, a subline of the murine myoblast cell line known for rapid differentiation and the formation of contractile microtubules associated with muscle-specific protein expression. The hMSCs dataset comprises of bone marrow-derived mesenchymal stem cells, a type of adult stem cell with multipotent differentiation potential. These cells, isolated from the mammalian bone marrow stroma, can differentiate into osteoblasts, chondrocytes, and adipocytes under appropriate *in vitro* or *in vivo* conditions. Experimental parameter settings are presented in Table 1.

**TABLE 1:** Experimental parameter setting.

Parameter	C2C12 dataset	hMSCs dataset
k	0.86	1.68
l	2	
$\beta_{u}$	30.99	27.45
$M_{p}$	276	209

To quantitatively assess the performance of various SC image segmentation methods, four standard evaluation metrics are employed: accuracy, recall, precision, and F1-score. The corresponding calculation formulas are provided in Equations 3–6.

$$\text{Accuracy}=\frac{TP+TN}{TP+TN+FP+FN}$$ (3)

$$\text{Recall}=\frac{TP}{TP+FN}$$ (4)

$$\text{Precision}=\frac{TP}{TP+FP}$$ (5)

$$F1=\frac{2*\text{Precision}*\text{Recall}}{\text{Precision}+\text{Recall}}$$ (6)

These metrics are derived from the confusion matrix, which quantifies classification performance. For a binary case with positive and negative classes, predictions are categorized as: True Positive (TP, correct positive prediction); False Positive (FP: incorrect positive prediction); False Negative (FN, incorrect negative prediction); True Negative (TN, correct negative prediction).

Cell confluency serves as a critical parameter for the evaluation of adherent cell cultures. It provides guidance for determining the appropriate timing for cell passaging and helps prevent overgrowth, thereby ensuring the maintenance of optimal culture conditions. Image processing techniques based on visual information analysis allow for automated and rapid estimation of cell confluency, as well as quantification of confluency error rate. The corresponding calculation formulas are provided in Equations 7, 8:

$$\text{confluency}=\frac{TP+FP}{\text{TP}+\text{FP}+\text{TN}+\text{FN}}$$ (7)

$$\text{error}=\left|{\text{confluency}}_{\text{method}}‐{\text{confluency}}_{\text{standard}}\right|$$ (8)

The standard method refers to the confluency obtained from cell staining images analyzed by CellProfiler from the same field of view, specifically the ratio of the area of stained cells to the total image area.

## 3 Results

After the image preprocessing steps, the accuracy of cell segmentation was clearly improved, as observed in Figure 1b, that indicates the preprocessing steps are essential.

**FIGURE 3:**
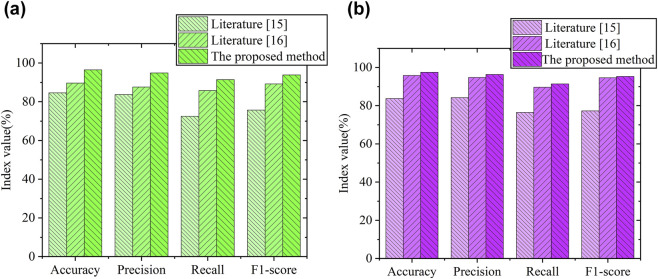
Comparison bar charts showing performance metrics, including accuracy, precision, recall, and F1-score. **(a)** Performance validation results of different methods on the hMSCs data set. **(b)** Performance validation results of different methods on the C2C12 data set.

The proposed method was evaluated against comparable approaches described in references and for SC image segmentation (Mota et al., 2021; Kusumoto and Yuasa, 2019). As seen in Figure 3, the proposed method achieved accuracy, recall, precision, and F1-score values of 96.5%, 94.9%, 91.4%, and 93.9%, respectively, on the hMSCs dataset. These metrics were consistently higher than those reported for the methods mentioned in the literature [15] and [16] (Mota et al., 2021; Kusumoto and Yuasa, 2019). On the C2C12 dataset, the proposed method yielded accuracy, recall, precision, and F1-score values of 97.5%, 96.3%, 91.4%, and 95.3%, respectively, again outperforming the other referenced methods. These findings indicate improved segmentation performance using the proposed approach.

**FIGURE 4:**
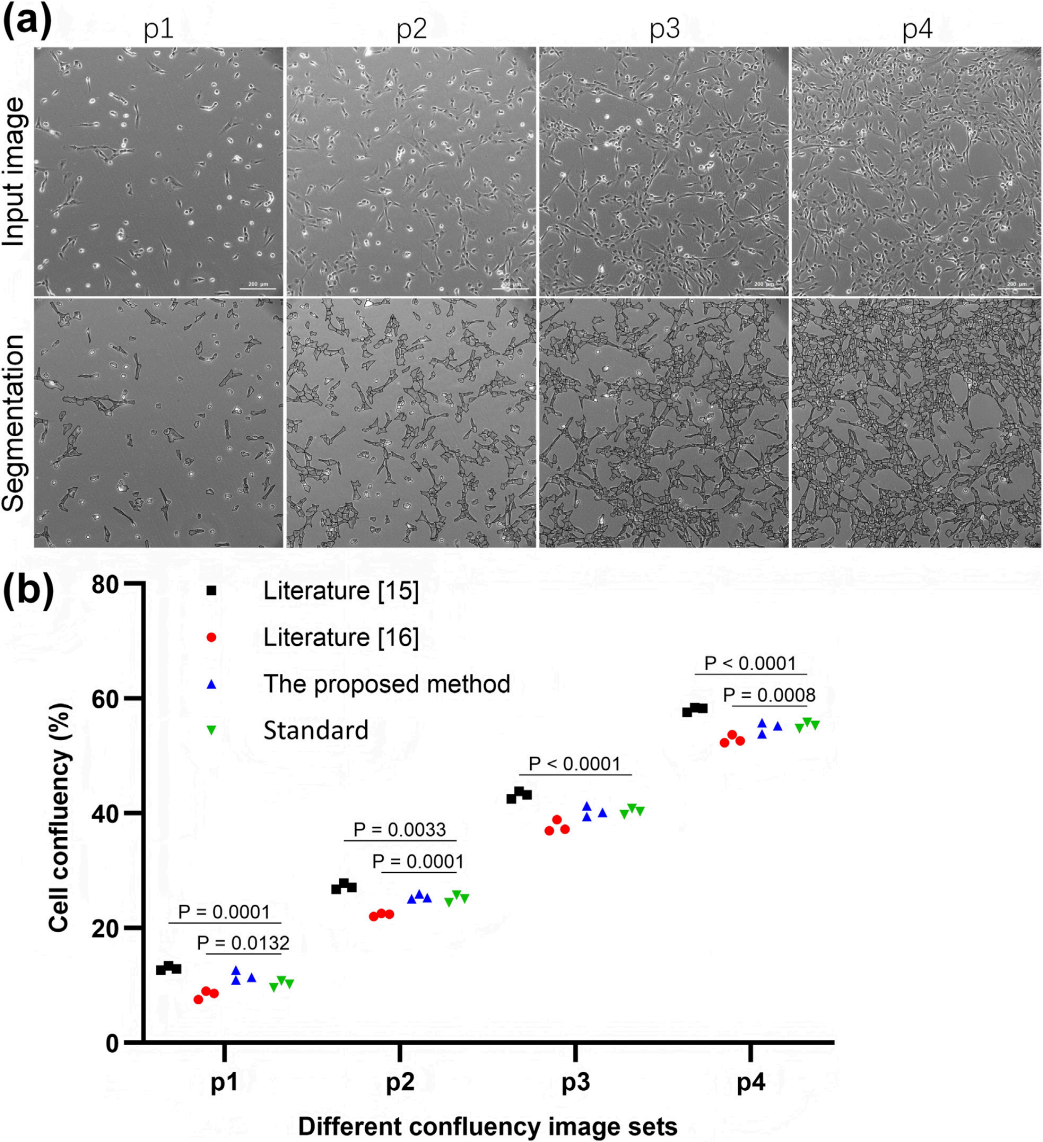
**(a)** Segmentation results of input images with varying cell confluency. (**b)** Comparison of confluency analyzed by different methods versus the standard method.

**FIGURE 5:**
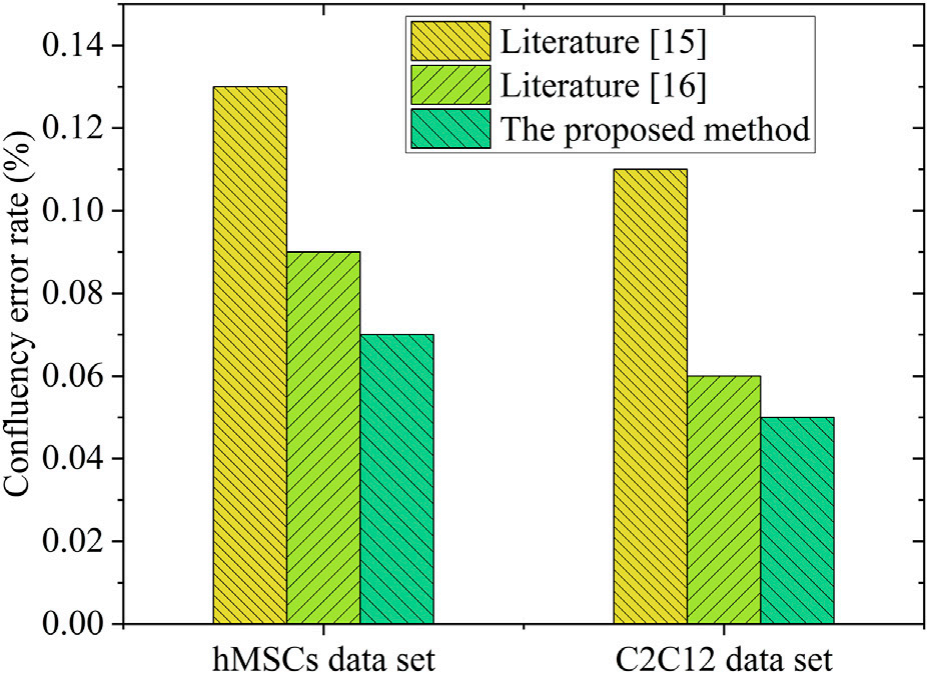
Bar chart comparing the confluency error rates for hMSCs and C2C12 data set, Three methods are shown: Literature [15], Literature [16] and the proposed method. The proposed method shows the lowest error rates for both data sets. Confluency error rate is measured in percentage on the vertical axis.

To further assess performance, four image sets (identified as p1, p2, p3, p4, each set contain three images with similar confluency) were selected for comparative analysis of the confluency and associated errors. Figure 4a shows representative segmentation results with varying confluency. And the analysis results are depicted in Figures 4b, 5.

According to Figure 4, the confluency values calculated using the proposed method were found to be closer to the reference standards compared to those obtained by the other methods, indicating greater accuracy. Figure 5 demonstrates that the confluency error rate on the hMSCs and C2C12 datasets was 0.07 and 0.05, respectively, representing the lowest error rates among the three evaluated methods. These outcomes further support the superior accuracy and robustness of the proposed method in SC image segmentation tasks.

## 4 Discussion

In medical image processing, cell image segmentation represents a critical step for applications such as cellular analysis, quantitative cell counting, and disease diagnosis. Conventional segmentation techniques frequently encounter difficulties related to variable illumination conditions, overlapping cellular structures, and noise interference, all of which can compromise segmentation accuracy. Although adaptive thresholding is widely applied for image segmentation tasks, its performance is often affected by fluctuations in illumination and the presence of noise (Roy et al., 2014). Additionally, halo artifacts—resulting from non-uniform illumination or imaging-related distortions—further complicate the segmentation process.

To address these limitations, advanced algorithms have been developed to enable more robust segmentation under complex imaging conditions. The adaptive thresholding module dynamically adjusts threshold values in response to local image features, such as pixel intensity and gradient variations, thereby improving resilience against illumination inconsistencies and noise interference (Xiying et al., 2025). The halo correction module is specifically designed to identify and compensate for halo artifacts. This is achieved by first detecting halo-affected regions using a combination of edge detection techniques and morphological operations. Subsequently, a corrective filter is applied to attenuate halo intensity, thereby enhancing the visibility and definition of cell boundaries (Kandel et al., 2018).

The experimental findings indicate that the SC image segmentation algorithm, which integrates adaptive thresholding with halo correction, demonstrates enhanced performance. These results validate its effectiveness in improving SC image processing and are consistent with recent advancements in segmentation methodologies across various biomedical imaging domains. Notably, the proposed method is a traditional image processing approach, distinguished by its lightweight implementation, high efficiency (processing a single image in 1–3 s), and full interpretability through explicit mathematical formulas and logical rules. This design aligns with the practical demand for rapid, transparent analysis in scenarios where computational resources are limited or interpretability of the segmentation mechanism is required—distinct from deep learning (DL) methods that typically rely on large-scale annotated datasets for training and involve high computational costs.

For example, Salvi et al. (2019) introduced “CARdiosphere,” a fully automated method for segmenting membranes and nuclei in cardiac spheroids (Salvi et al., 2019). This method achieved high segmentation accuracy for pericardial membranes and performed at an expert level in cell nuclei detection which was evaluated on 1,160 three-dimensional cardiac spheroid datasets. This emphasizes the significance of automation and precision in analyzing complex biological structures, which aligns with the objectives of adaptive thresholding in SC image analysis.

Similarly, Scherr et al. (2020) proposed a novel boundary representation strategy for segmenting touching cells in microscopy images (Scherr et al., 2020). This method, based on distance-inspired boundary modeling, improved segmentation accuracy on training datasets by addressing edge ambiguity—an issue comparable to halo artifacts observed in SC images. The halo correction mechanism implemented in the present SC segmentation algorithm addresses similar challenges by refining boundary clarity through adaptive filtering and potentially multi-scale analysis.

In medical diagnostics, additional technical advancements have been reported. Shi et al. (2023) developed a multi-strategy-driven slime mould algorithm (RWGSMA), which enhanced segmentation accuracy and efficiency in lupus nephritis imaging (Shi et al., 2023). This was achieved through the integration of a random backup strategy, a dual adaptive weighting strategy, and a rank-based search strategy. RWGSMA demonstrated superior convergence speed and global optimization performance in IEEE CEC2017 benchmark testing. Its effectiveness in multi-threshold segmentation was validated using PSNR, SSIM, and FSIM metrics, with notable resistance to local optima in threshold ranges spanning 3 to 20 levels.

Furthermore, Mancini et al. (2024) investigated the effects of adaptive versus fixed thresholding strategies in coronary computed tomography angiography across different platforms (Mancini et al., 2024). Their findings indicated that adaptive thresholding, when adjusted according to individual vascular characteristics, minimized segmentation errors arising from inter-patient variability or image quality inconsistencies. This approach provided more accurate assessments of plaque vulnerability, whereas fixed thresholds were associated with overestimation or underestimation of calcified or lipid-rich components.

Despite significant advancements in microscopy technologies, conventional analysis methods face critical limitations. One of the primary concerns is the subjectivity and limited reproducibility associated with manual interpretation. For instance, in tumor-stroma ratio assessment, inter-observer variability among pathologists exceeded 20% (Kazemi et al., 2023). Additionally, threshold-based software tools, such as ImageJ, encounter challenges in complex imaging conditions, including overlapping cells, uneven staining, and background noise. In clinical contexts such as HER2 immunohistochemical scoring, ambiguous intensity gradients frequently lead to diagnostic inconsistencies, which can affect subsequent therapeutic decision-making (Chen et al., 2025).

Low throughput also represents a major obstacle, particularly for high-content drug screening or large-scale clinical applications. Manual analysis of imaging plates can require several hours, whereas automated computational tools significantly reduce processing time to within minutes. These limitations underscore the pressing need for advanced, intelligent image analysis technologies. Given the small size of our dataset (only 50 images per dataset, i.e., C2C12 and hMSCs datasets), which is insufficient to train DL models to fully leverage their feature-learning advantages, and the distinct design objectives between our method and DL approaches, we did not include DL baselines in the comparative experiments. Instead, the focus of this study is to explore the optimized potential of traditional image processing techniques in stem cell image segmentation, providing a practical alternative for scenarios where DL methods are not feasible due to data or resource constraints. We acknowledge that a comprehensive comparison with mature DL methods will be a valuable direction for future research with larger annotated datasets.

## 5 Conclusion

This study first examined the principles underlying the cognitive mechanism of visual information processing. Building upon this foundation, the limitations of conventional SC image segmentation methods were analyzed using phase contrast microscopy images. An optimized image segmentation approach incorporating halo correction was subsequently proposed and experimentally validated.

The proposed method demonstrated improved segmentation performance compared to similar existing techniques. However, there are certain limitations, nevertheless. Specifically, for SC images exhibiting high confluency, the segmentation accuracy requires further enhancement. Addressing this challenge represents a key direction for future research work.

## Data Availability

The raw data supporting the conclusions of this article will be made available by the authors, without undue reservation.

## Abbreviations

SC, stem cell; hMSCs, Human Mesenchymal Stem Cells; RWGSMA, multi-strategy-driven slime mould algorithm; LN, lupus nephritis; CCTA, coronary computed tomography angiography; TSR, tumor-stroma ratio; PSNR, Peak Signal-to-Noise Ratio; SSIM, structural similarity index measure; FSIM, feature similarity index mersure; IEEE, Institute of Electrical and Electronics Engineers; CEC, Congress on Evolutionary Computation.
